# Effects of force- and velocity-oriented conditioning activities on jump height in strength-deficient male participants

**DOI:** 10.3389/fphys.2025.1545621

**Published:** 2025-03-04

**Authors:** Dawid Koźlenia, Žiga Kozinc, Amador Garcia-Ramos, Jarosław Domaradzki

**Affiliations:** ^1^ Faculty of Physical Education and Sport, Wroclaw University of Health and Sport Sciences, Wroclaw, Poland; ^2^ Faculty of Health Sciences, University of Primorska, Izola, Slovenia; ^3^ Department of Physical Education and Sport, Faculty of Sport Sciences, University of Granada, Granada, Spain; ^4^ Department of Sports Sciences and Physical Conditioning, Faculty of Education, Universidad Catolica de la Santisima Concepción, Concepción, Chile

**Keywords:** post-activation performance enhancement, force–velocity profile, power, squat jump, men

## Abstract

**Introduction:**

Various factors are known to influence the effectiveness of post-activation performance enhancement (PAPE) protocols. However, whether individual’s force–velocity (FV) profile affects the impact of conditioning activity (CA) remains unclear. This study examined whether PAPE is influenced by addressing individual strength deficits, identified through FV profiling, using either force- or velocity-oriented conditioning. Specifically, we (i) assessed the effectiveness of force-oriented (PAPE-F) and velocity-oriented (PAPE-V) protocols on acute jump height (JH) performance in individuals with strength deficits and (ii) investigated whether the magnitude of force–velocity imbalance (FV_imb_) is significantly associated with PAPE in JH.

**Methods:**

Twenty-five young (19–27 years), resistance-trained male individuals (≥2 years of continuous training) who exhibited a strength deficit, determined by FV_imb_ in the squat jump (SJ), were included in this study. They performed either three sets of five assisted jumps (PAPE-V; load reduced by 30% of body mass) or three four-second sets of maximal isometric contractions (PAPE-F), each with 1 min rest intervals. JH was measured at baseline and 3, 6, and 9 min post-CA.

**Results:**

A three-way (group × condition × time) repeated measures ANOVA revealed significant effects of time (F = 7.78; partial-η^2^ = 0.14; p < 0.01) and a significant condition × time interaction (F = 16.57; partial-η^2^ = 0.26; p < 0.01) for JH. The Bonferroni *post hoc* test revealed significant within-group improvements after PAPE-F at the 6th min (p < 0.01; ES = 0.32) and 9th min (p < 0.01; ES = 0.33) compared to baseline and after PAPE-V at the 3rd min (p < 0.01; ES = 0.24), 6th min (p < 0.01; ES = 0.36), and 9th min (p < 0.01; ES = 0.30) compared to baseline. Linear regression models showed that individuals with lower FV_imb_ exhibited greater PAPE effects following the PAPE-F protocol (β = 0.63; R^2^ = 40; p = 0.03), but no significant associations were observed between these two variables for the PAPE-V protocol (R^2^ = 0.19; p = 0.53).

**Discussion:**

These results suggest that individuals can achieve comparable acute JH improvements using force- or velocity-oriented CAs, although force-oriented CA may promote greater gains in individuals with lower FV_imb_.

## 1 Introduction

Force and velocity are the two key components of mechanical power output (Cormie et al., 2011a, 2011b). In many sports, the ability to achieve high levels of mechanical power is a critical determinant of physical performance, particularly in activities such as jumping and sprinting (Cronin and Sleivert, 2005; Cronin and Hansen, 2005; Pupo et al., 2020). However, it is important to note that the effectiveness of these explosive movements is not solely dictated by maximal power output (P_max_) but also by the optimization of the force–velocity (FV) profile. Specifically, an optimal FV profile in jumping is achieved when an individual generates P_max_ during bodyweight jumps, performed without any external resistance or assistance. Individuals who generate P_max_ under loading conditions greater than bodyweight jumps exhibit a force-oriented profile (i.e., velocity deficit), while those who generate P_max_ under loading conditions lighter than bodyweight jumps display a velocity-oriented profile (i.e., strength deficit) (Samozino et al., 2012; Samozino et al., 2022).

Mechanical power can be acutely increased through a phenomenon known as post-activation performance enhancement (PAPE) (Seitz and Haff, 2016; Koźlenia and Domaradzki, 2023a; 2023b). This strategy typically involves performing a short, high-intensity conditioning activity (CA) immediately before the target effort. However, moderate- to high-intensity conditioning activities (CAs), ranging from 60% 1RM to above 90% 1RM, have also been reported to induce PAPE responses performed in a single set (Krzysztofik et al., 2021), across multiple sets (Lesinski et al., 2013; Wilson et al., 2013; Seitz and Haff, 2016), or regardless of the total number of sets (Dobbs et al., 2019; Garbisu-Hualde and Santos-Concejero, 2021). In addition, these responses typically manifest between 3 and 10 min following the CA (Gouvêa et al., 2013; Lesinski et al., 2013; Wilson et al., 2013; Seitz and Haff, 2016; Garbisu-Hualde and Santos-Concejero, 2021). Rassier and Macintosh (2000) postulated that PAPE is the product of heightened excitation and reduced fatigue. After a possible transient performance decline, a periodic improvement in desired parameters is observed (Boullosa, 2021). This phenomenon occurs through an increase in body temperature, improved intracellular fluid flow, and enhanced neural-muscle impulse transmission and is simultaneously inhibited by fatigue (Blazevich and Babault, 2019; Fischer and Paternoster, 2024). The PAPE effect can be strategically utilized both during training and immediately prior to sports competitions (Đurović et al., 2022) making this approach a valuable part of warm-up routines (Bishop, 2003). Although many studies have shown improvements in individual indicators of physical performance (e.g., jump height [JH] and sprint time) through various CA, the magnitude of these effects often depends on individual characteristics (e.g., strength levels or training experience) and the specific attributes of the PAPE protocols (modality, intensity, rest interval, etc.) (Seitz and Haff, 2016).

There is a need to deepen the analysis of individual factors influencing the effectiveness of the PAPE effect (Seitz and Haff, 2016; Baena-Raya et al., 2022; Kasicki et al., 2024). A key aspect under consideration is the individual’s motor characteristics, specifically their FV profile (Morin and Samozino et al., 2016; Jiménez-Reyes et al., 2017). Given the assumption that an optimal combination of maximal force (*F*_0_) and maximal velocity (*v*_0_) exists to maximize JH performance for a given level of P_max_ (Samozino et al., 2008), it is plausible that individuals with a strength deficit or a velocity deficit would benefit more from CA specifically targeting *F*_0_ and *v*_0_, respectively. However, the specific effects of different CAs (e.g., force- or velocity-oriented) on the magnitude of PAPE in athletes with different FV characteristics remain unexplored. The method for determining the vertical FV profile, as proposed by Samozino et al. (2016), Morin and Samozino et al. (2016), has been successfully implemented in physical preparation across various sports disciplines. Despite the effective use of the FV profile in the physical training process (Jiménez-Reyes et al., 2017), there is currently a lack of studies incorporating the individual FV profile in the implementation of PAPE protocols to optimize physical performance. The findings of Baena-Raya et al. (2022) indicate that FV profile parameters are strongly associated with various sports performance metrics and can be altered through short-term training programs (Nishioka and Okada, 2022). A reduction in force–velocity imbalance (FV_imb_) may be associated with improvements in physical performance (Jiménez-Reyes et al., 2017), thus suggesting that individuals with a more balanced force–velocity profile could experience more pronounced enhancements following CA. Further research is needed to build on these findings and establish practical guidelines for incorporating the FV profile when choosing CA for inducing PAPE.

This study aimed to investigate whether PAPE effects are influenced by addressing individual strength deficits detected by the analysis of the FV profile through force- or velocity-oriented conditioning activities. Specifically, we aimed (i) to assess the effectiveness of force-oriented (PAPE-F) and velocity-oriented (PAPE-V) conditioning activities on acute changes in JH performance in individuals with a strength deficit and (ii) to elucidate whether the magnitude of FV_imb_ is significantly associated with the magnitude of PAPE in JH. We hypothesized that the PAPE-F protocol would be more effective than the PAPE-V protocol as we intentionally selected male participants with a strength deficit, whereas individuals with greater FV_imb​_ were expected to exhibit smaller PAPE effects with both protocols. The obtained results could offer practitioners more insights for optimizing the application of CA, tailored to the individual characteristics of the FV profile, to acutely enhance jump performance during training sessions or competitions.

## 2 Material and methods

### 2.1 Study design

This study conducts a randomized controlled trial with parallel groups (allocated to force- and velocity-oriented CA) and repeated measures with a cross-over between PAPE and control session. In the pre-screening, during recruitment, participants provided details about their training background, barbell high-bar back squat one-repetition maximum (1RM), and injury history with a survey. During the initial meeting, participants underwent a body morphology assessment (body mass and height and lower limbs length) and then completed a standard warm-up, which included a 5-min treadmill walk at 6 km/h, dynamic joint mobilizations, three sets of 12–15 repetitions of bodyweight squats, and two sets of lunges with an empty bar. Afterward, a few squat jumps were performed to familiarize the participants with the testing requirements.

The FV profile assessment was performed using the two-point method with squat jumps (SJs). To maintain consistency in movement patterns, participants performed squat jumps using an empty stick (0.5 kg) and a bar loaded with an additional 70% of their body mass. Subsequently, the participants’ 1RM was verified using the individual load-velocity method. Following this, participants were familiarized with both PAPE protocols. In the force-based CA (PAPE-F), participants performed three maximal isometric contractions against an immovable bar, each lasting 4 s, with 1-minute rest intervals. The height of the bar was positioned to 90° at the knee level, corresponding to the squat jump position. Participants positioned themselves under the bar as they would for a back squat, and after receiving a signal from the research team, they pushed as hard as they can with constant encouragement from researchers.

In the velocity-based CA (PAPE-V), they completed five assisted vertical jumps using resistance bands set at 30% of body weight, also with 1 min of rest intervals. After the familiarization session, participants were randomly assigned to either the PAPE-force (PAPE-F) or PAPE-velocity (PAPE-V) group. During the two experimental sessions (PAPE and control), participants in both groups followed the same standard warm-up, as previously described. Baseline squat jump measures were then performed. Following this, participants either performed the appropriate conditioning activity (due to group adherence) or completed a control condition consisting of a 4-min treadmill run at 6 km/h. Both sessions were performed in a counterbalanced order. Squat jumps were subsequently performed again at 3-, 6-, and 9-min post-conditioning activity. JH [cm] was analyzed as the primary outcome (Xu et al., 2025).

### 2.2 Participants

The required sample size (Faul et al., 2007; Kang, 2021) for the adopted statistical analysis (F-tests, repeated measures, and within-between interactions) was determined through a power analysis conducted using GPower software (version 3.1.9.6). The analysis was designed to achieve 80% statistical power to detect a minimum effect size (ES) of 0.3, with a significance level set at 0.05 (Rhea, 2004) and a correlation of 0.5 between repeated measures, indicating a minimum requirement of 18 participants. Finally, the study sample was n = 25, and *post hoc* sample size calculations indicated that for n = 25, with a power of 0.80 and α = 0.05, ES was 0.24.

Before the start of the project, participants were required to complete a survey to provide data relevant to the study’s inclusion and exclusion criteria. The inclusion criteria were established as a minimum of 2 years of engagement in continuous resistance training, male individuals with a minimum chronological age of 18 years, and a barbell back squat 1RM ≥ 120% of body mass. The exclusion criteria were musculoskeletal injury within 4 weeks before the measurements and any medical contraindications in undertaking physical effort. Initially, 31 subjects were identified, but due to a lack of appropriate strength level (n = 2) and a lack of availability during testing days (n = 4), six participants were excluded. In the present study, the participants were individuals exclusively focused on strength training without engaging in professional-level preparation in any sports discipline. At the same time, they were not restricted from taking part in other forms of recreational physical activity. Detailed characteristics of the 25 participants included in our study are presented in Table 1.

**TABLE 1:** Descriptive characteristics of the study sample.

Variable	PAPE-F (n = 12)	PAPE-V (n = 13)	t	p	ES
	Mean ± SD (95% CI)	Mean ± SD (95% CI)			
Age [years]	23.8 ± 3.0 (21.9–25.7)	24.1 ± 2.8 (22.5–25.8)	−034	0.73	0.10
Body height [cm]	182.7 ± 6.4 (178.6–186.8)	184.7 ± 22.6 (171.1–198.4)	−0.30	0.77	0.12
Body mass [kg]	76.6 ± 7.3 (72.0–81.2)	79.3 ± 11.6 (72.3–86.3)	−0.69	0.50	0.27
Body mass index [kg/m^2^]	22.9 ± 1.4 (22.0–23.8)	23.7 ± 3.8 (21.4–25.9)	−0.64	0.53	0.25
Back squat 1RM [kg]	119.6 ± 15.4 (109.8–129.4)	127.1 ± 22.2 (113.7–140.9)	−0.98	0.34	0.39
Relative strength [body mass/back squat]	156.9 ± 15.3 (146.54–166.0)	159.8 ± 10.3 (153.6–166.0)	−0.68	0.50	0.27
Gym experience [years]	4.0 ± 1.5 (3.0–5.0)	4.9 ± 2.0 (3.0–5.8)	−0.11	0.91	0.04
Weekly training volume [min]	324.2 ± 128.4 (242.5–405.8)	330.0 ± 103.9 (267.2–392.8)	−0.13	0.90	0.05
*F*_0_ (N/kg)	42.2 ± 5.7 (38.6–45.9)	49.4 ± 6.7 (45.4–53.4)	−2.88	0.01*	1.15
*v*_0_ (m/s)	3.09 ± 0.47 (2.79–3.38)	3.28 ± 0.53 (2.97–3.60)	−0.99	0.33	0.37
*P*_max_ (W/kg)	33.0 ± 7.9 (27.9–38.0)	40.4 ± 7.3 (36.0–44.8)	−2.45	0.02*	0.97
*Sfv*	−13.8 ± 1.9 (−15.0–−12.6)	−15.4 ± 2.9 (−17.2–−13.6)	1.58	0.13	0.63
FV_imb_ (%)	79.8 ± 14.2 (70.8–88.8)	72.8 ± 14.5 (64.1–81.6)	1.22	0.24	0.48

*Statistically significant difference (p < 0.05) obtained by the student t-test comparison for independent sample.

### 2.3 Body morphology

Body height was measured using a standard anthropometer (Swiss Anthropometer, GPM Anthropological Instruments, DKSH Ltd., Zürich, Switzerland), and body mass was assessed using the InBody230 device (InBody Co., Ltd., Cerritos, CA, United States), reliability of which was confirmed (McLester et al., 2020). Participants stood barefoot with their heels together, back straight, and head in the Frankfort horizontal plane. The measurement for body height was recorded to the nearest 0.1 cm, and body mass was recorded to the nearest 0.1 kg to ensure accuracy. All measurements were performed according to principles based on standards established by The International Society for the Advancement of Kinanthropometry (ISAK) (Marfell-Jones et al., 2006). Participants were instructed to avoid any intense physical activity and refrain from eating or drinking for at least 3 h before the measurements. They were also asked to empty their bladders immediately prior to the assessment. Body mass index (BMI) was calculated using the formula: BMI = body mass [kg] divided by height squared [m^2^].

### 2.4 Force–velocity profiling

The force–velocity profile was determined using the two-point method (Garcia-Ramos and Jaric, 2018; García-Ramos et al., 2021). The required parameters were body mass, lower limb length with all joints fully extended, and the height of the lower limbs at 90° in the knees, measured as the vertical distance from the greater trochanter to the floor. Squat JH was performed using a wooden stick (0.5 kg) with an additional load and a load equal to 70% of body mass. The minimum required for reliability JH was 10 cm (Janicijevic et al., 2020; Garcia-Ramos and Jaric, 2018). The calculated parameters were as follows.

*F*_0_ (N/kg)—It represents the theoretical maximal force the lower limbs can produce during a ballistic push-off, calculated from the y-intercept of the FV relationship in loaded jump squats. It reflects the maximal concentric force per unit of body mass, offering a comprehensive measure of force capacity beyond a single lift, such as a squat 1RM.

*v*_0_ (m/s)—It is the theoretical maximum velocity of the lower limbs during ballistic push-off, derived from the x-intercept of the FV relationship. This value represents the ability to generate force at very high velocities, which is nearly impossible to measure directly.

*P*_max_ (W/kg)—It is the maximum mechanical power output of the lower limbs, calculated using P_max_ = F0 × V0/4 or the peak of the power–velocity relationship. It indicates the athlete’s ability to produce power during concentric and ballistic movements.

*Sfv*—The slope of the FV relationship represents the balance between force and velocity capabilities. A steeper (more negative) slope indicates a more force-oriented profile, while a flatter slope indicates a velocity-oriented profile.

*Sfv*_opt_—The specific slope of the force–velocity relationship maximizes the jump height for a given push-off distance, body mass, and maximal power (P_max_). It reflects the ideal balance between force and velocity for an individual’s ballistic push-off.

FV_imb_ (%)—It is the percentage difference between an individual’s actual FV profile (*Sfv*) and their optimal profile (Sfv_opt_). A value of 100% indicates a balanced profile, while values above or below signify imbalances, with deficits in either force or velocity.

### 2.5 One-repetition maximum in back squat (high bar) verification

To establish the 1RM for the full back squat, the Vitruve linear position transducer (Vitruve, SPEED4LIFTS S.L., Madrid, Spain) was utilized, leveraging the relationship between load and velocity, as described in prior research (Jidovtseff et al., 2011; Signore, 2021). This method ensures both accuracy and safety, supported by the device’s proven reliability (Martínez-Cava et al., 2020). Participants first performed a maximal deep back squat with an unloaded bar to determine their individual maximum depth while maintaining proper technique and safety. This depth was consistently used throughout the 1RM protocol and all subsequent conditioning activities, verified by parallel markers adjusted to each participant’s height. An investigator supervised each session to ensure consistency and proper execution. The 1RM back squat protocol began with a specific warm-up, which included a set of 12–15 repetitions of back squats using an empty bar, followed by a set of 10–12 repetitions performed at a mean velocity of 1.0–1.2 m/s. Next, participants performed 2–3 repetitions per set at a mean velocity of 1.0–0.75 m/s. When the mean velocity decreased to 0.75–0.5 m/s, participants completed two repetitions per set. Once the mean velocity fell below 0.5 m/s, sets were reduced to a single repetition. The protocol allowed for 3–5 min of rest between sets, with individuals choosing the exact rest duration based on personal preference. The load was increased by 5%–10% after each set, with participants deciding the increment based on their perceived readiness. No more than five sets were permitted within each velocity range. After a rest period of 2–3 min, participants continued until failure, defined as either the inability to complete the lift or failure to achieve the established squat depth.

### 2.6 Conditioning activity protocols

The force-oriented PAPE protocol (PAPE-F) involved three 4-second sets of maximal isometric contractions evoked by pushing as strongly as possible on an immovable bar, with a-minute rest interval. Optimal performance enhancement has been associated with repeated isometric stimulations of 3–5 s (French et al., 2003). The height of the bar was positioned at 90° to the knees, similar to the position established for squat jumps. Participants positioned themselves under the bar as they would for a back squat, and after receiving a signal from the research team, they pushed as hard as they could with constant encouragement from the researcher.

**FIGURE 1:**
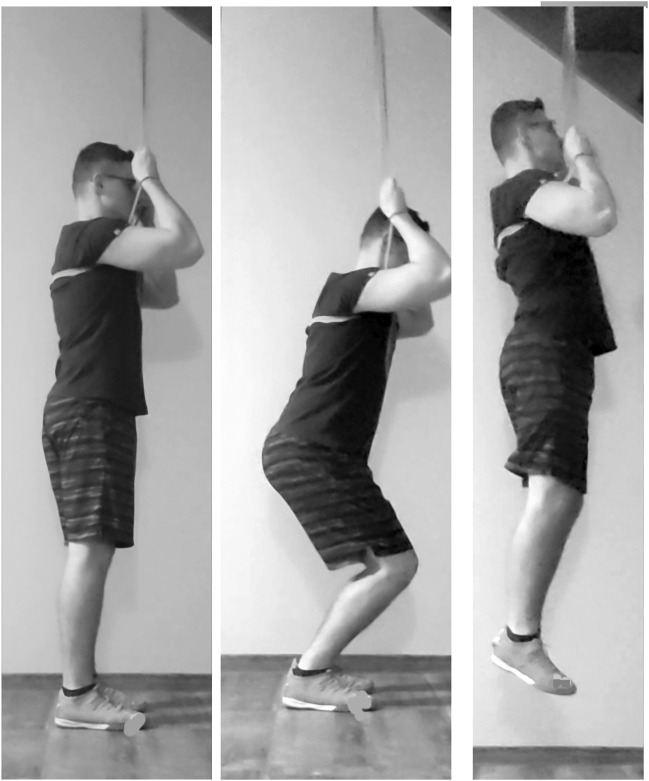
Setup for assisted band jumps performed as velocity-oriented CA.

The velocity-oriented PAPE protocol (PAPE-V) was utilized, with five vertical jumps with a resistance band (assisted band jump) with 1 minute of rest (Koźlenia and Domaradzki, 2024). The band resistance was accommodated on 30% of body weight. The assisted band jump protocol was adapted from the method outlined by Wilson and Kritz, (2014). Participants began in a standing position with an elastic power band (Just7gym, Wilkszyn, Poland) suspended overhead. To determine the correct attachment height and ensure the desired load reduction—targeting a 30% decrease in body weight during the downward phase before the jump—a weight equivalent to 30% of the participant’s body weight was attached to the band at a specific height (Tran et al., 2012; Markovic et al., 2011; Strate et al., 2022). Once the appropriate band and attachment height were established, the participants positioned the band under their armpits, held it with their hands, and performed a series of five assisted band jumps (Figure 1).

### 2.7 Jump height measures

The flight time of the SJ was quantified using a validated contact mat (Chronojump, Barcelona, Spain) and subsequently used to calculate JH according to the following equation: JH = (9.81 × (flight time)^2^/8). Chronojump demonstrated very high reliability (α = 1.00; CV = 4.28 ± 1.95%). The smallest worthwhile change (SWC) was 1.3 cm, with a typical error (TE) of 0.29 cm, leading to a signal-to-noise ratio of 4.5. These findings indicate that Chronojump can accurately and consistently detect meaningful changes in vertical jump performance (Pueo et al., 2020). Between the contact mat and the computer running measurement software, the Chronopic device acts as an intermediary, featuring a sampling frequency of 1,000 Hz. The test was performed as described by Comfort et al. (2018). Participants began in a static squatting position with their knees bent at approximately 90° and hands placed on their hips to minimize arm movement. Without any preliminary countermovement or dip, they were instructed to jump vertically as high as possible from this stationary position. The position had to be held 3 s before jumping. This method eliminates the stretch-shortening cycle, focusing solely on concentric muscle action. The measurements were performed at baseline and then after the CA protocol at baseline and 3rd, 6th, and 9th min, with one maximal attempt at each time point. The analyzed parameters were changes (Δ) in absolute = Jump best–baseline [cm] and relative = (Absolute Δ/baseline) *100%.

### 2.8 Statistics

The normality of data distribution was assessed using the Shapiro–Wilk test and a significance level of α = 0.05. The results are presented as the mean ± standard deviation (SD), with accompanying 95% confidence intervals (CIs). The Student’s t-test for an independent sample was performed to assess descriptive characteristics between groups. Levene’s test was utilized to verify the homogeneity of variances, while Mauchly’s test was used to check for data sphericity. The data homogeneity was confirmed at α = 0.05, whereas data sphericity was violated for the time factor (W = 0.74; p = 0.02), and the results were reported with the Greenhouse–Geisser correction (ε = 0.87). A three-way repeated-measures analysis of variance (ANOVA) was performed to analyze JH values, with one between-participant factor–group (PAPE-F and PAPE-V) and two within-participant factors–condition (intervention and control) and time (baseline, 3 min, 6 min, and 9 min). Effect sizes were calculated using partial eta squared (ηp^2^) and categorized as small (0.01 ≤ ηp^2^ < 0.06), medium (0.06 ≤ ηp^2^ < 0.14), or large (ηp^2^ ≥ 0.14). Significant results indicated by the F-ratio prompted further analysis using *post hoc* Bonferroni tests to identify specific differences in JHs over time. Cohen’s d effect size (ES) was calculated and categorized as trivial/negligible (ES < 0.2), small (0.2 ≤ ES < 0.5), moderate (0.5 ≤ ES < 0.8), and large (ES ≥ 0.8) (Cohen et al., 2003). Linear regression analyses were performed to examine the relationship between JH expressed as relative percentages (%) and FV_imb_. Relative strength was also included in the linear regression model to assess its possible contribution to the CA effects. In addition, a multivariate regression analysis incorporating both FV_imb_ and relative strength was conducted to verify the unique predictive value of FV_imb_. The calculated parameters were beta (β), a standardized regression coefficient indicating the relative importance of each predictor in the model; R^2^ representing the proportion of variance in the dependent variable explained by the regression model; and standard error (SE) reflecting the average distance by which observed values deviate from the model’s predicted values. The results for *p*-values less than 0.05 were considered statistically significant for all tests, which were carried out using Statistica 13.0 software (StatSoft Poland, Krakow, Poland).

## 3 Results

Baseline data of individuals included in PAPE-F and PAPE-V groups are presented in Table 1. Significant differences were only observed for *F*_0_ and *P*_max_, with the PAPE-V group showing greater values than the PAPE-F group (ES = 1.15 and 0.97, respectively).

Table 2 presents JHs in consecutive time points, with relative change (Relative Δ [%]) calculated as (best post-intervention results−baseline/baseline) *100% for both groups, considering Alco control conditions.

**TABLE 2:** Jump height measures during squat jump after conditioning activity and control conditions.

Group	Time	Jump height (cm) Mean ± SD (95% CI)	
		Experimental condition (PAPE)	Control condition (no PAPE)
Force-oriented PAPE	Baseline	37.2 ± 5.2 (33.9–40.5)	37.3 ± 5.2 (34–40.6)
	3rd min	37.1 ± 4.7 (34.1–40.1)	37.4 ± 4.8 (34.4–40.5)
	6th min	38.9 ± 4.9 (35.8–42.1)	36.9 ± 4.8 (33.8–40)
	9th min	38.9 ± 4.5 (36.1–41.7)	37.4 ± 5.3 (34–40.7)
	Relative Δ [%]	6.6 ± 5.4 (3.1–10)	1.9 ± 1.9 (0.7–3.2)
Velocity-oriented PAPE	Baseline	37.6 ± 6.6 (33.6–41.6)	37.2 ± 6.4 (33.4–41.1)
	3rd min	39.3 ± 7.5 (34.8–43.8)	37 ± 6.4 (33.1–40.8)
	6th min	40.1 ± 7.3 (35.7–44.5)	36.7 ± 7.1 (32.4–41)
	9th min	39.8 ± 8 (35–44.7)	36.7 ± 6.7 (32.7–40.8)
	Relative Δ [%]	8.3 ± 4 (5.8–10.7)	1.5 ± 1.3 (0.6–2.3)

The three-way repeated measures ANOVA revealed a significant effect of time (F = 7.78; partial-η^2^ = 0.14; p < 0.01) and an interaction of time × condition (F = 16.57; partial-η^2^ = 0.26; p < 0.01), with a lack of statistically significant effect of the group (F = 0.06; partial-η^2^ = 0.01; p = 0.81), condition (F = 0.81; partial-η^2^ = 0.02; p = 0.37), and other interactions group × condition (F = 0.2; partial-η^2^ = 0.01; p = 0.66), group × time (F = 1.21; partial-η^2^ = 0.03; p = 0.31), and group × time × condition (F = 2.25; partial-η^2^ = 0.05; p = 0.08).

Further one-way repeated measures ANOVAs revealed that statistically significant improvements in JH were present in experimental sessions both in PAPE-F (F = 8.52; partial-η^2^ = 0.47; p < 0.01) and PAPE-V (F = 11,20; partial-η^2^ = 0.48; p < 0.01) groups. Conversely, no statistically significant effects were noted in control sessions for both groups (F = 1.01; partial-η^2^ = 0.09; p = 0.40, and F = 1.09; partial-η^2^ = 0.09; p = 0.36, respectively).

**FIGURE 2:**
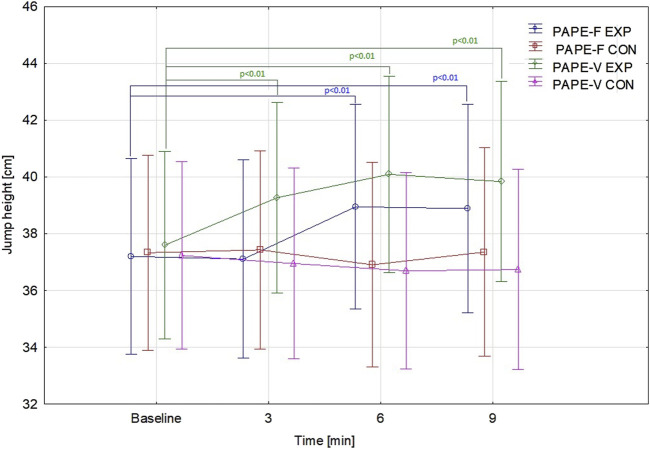
Jump height results over time according to the type of conditioning activity and control conditions. Points represent means. Bars represent 95% confidence intervals. Abbreviations: PAPE-F, group performed force-oriented conditioning activity; PAPE-V, group performed velocity-oriented conditioning activity; EXP, experimental settings; CON, control settings.

A detailed *post hoc* comparison with Bonferroni correction revealed intra-group differences in repeated measures in both types of conditioning activity (p < 0.01), meaning that both protocols were effective for increasing JH. The statistically significant improvement compared to baseline was visible in the PAPE-V group at 3rd min (p < 0.01; ES = 0.24), 6th min (p < 0.01; ES = 0.36), and 9th min (p < 0.01; ES = 0.30). However, in the PAPE-F group, the JH enhancement toward baseline was observed later, at 6th min (p < 0.01; ES = 0.32) and 9th min (p < 0.01; ES = 0.33). In addition, the results measured at 6th and 9th min were higher than those at the 3rd min (p < 0.01; ES = 0.37; and p < 0.01; ES = 0.39, respectively) (Figure 2).

**FIGURE 3:**
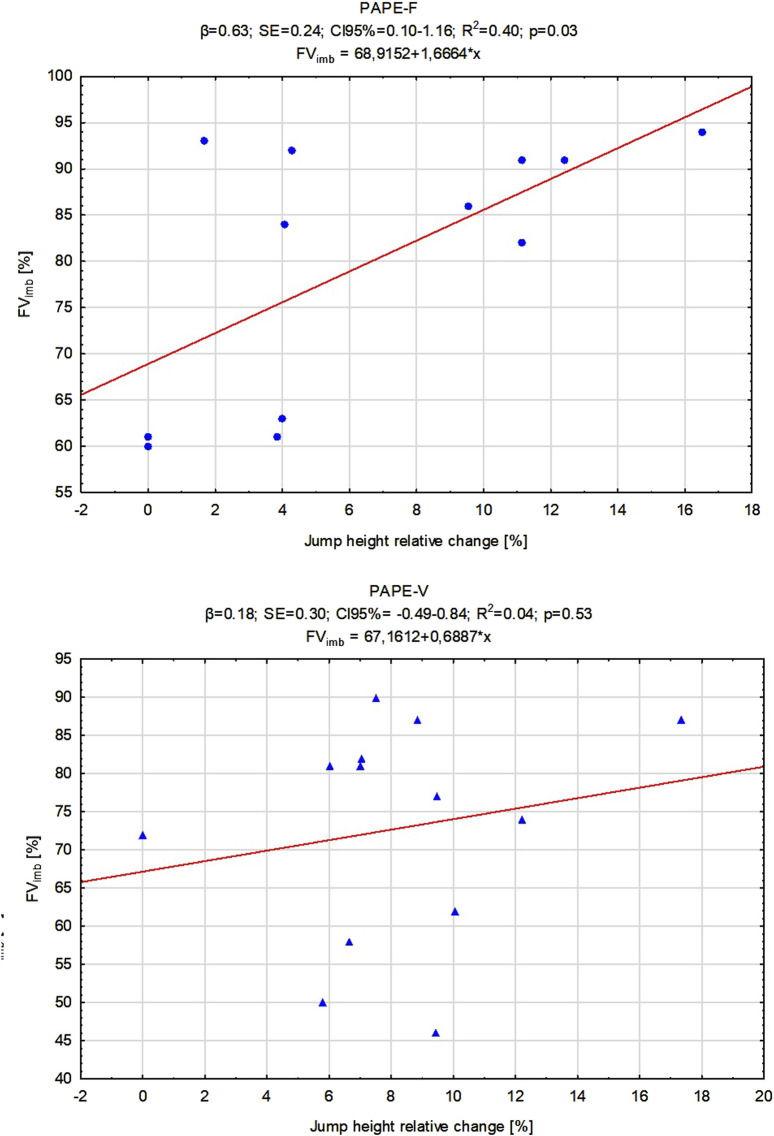
Linear regression models for jump height changes after conditioning activities related to individual force–velocity imbalance (FVimb%). Abbreviations: PAPE-F, group performed force-oriented conditioning activity; PAPE-V, group performed velocity-oriented conditioning activity; β indicates the strength and direction of the relationship between independent variables and the dependent variable; SE, standard error; CI 95%, confidence interval; R^2^, coefficient of determination measures how well the regression model explains the variance in the dependent variable.

Regression analysis showed that individuals with lower FV_imb_ exhibited greater PAPE effects following the PAPE-F protocol (p = 0.03), but no significant associations between these two variables were observed for the PAPE-V protocol (Figure 3). To determine whether FV_imb_ provides unique predictive value beyond overall strength, we first ran linear regressions using strength level as the predictor of changes in jump performance after CA. The results showed no statistically significant effects for PAPE-F (β = 0.21, SE = 0.30, R^2^ = 0.04, p = 0.50) or PAPE-V (β = 0.16, SE = 0.29, R^2^ = 0.02, p = 0.58). Next, we tested both CA protocol groups using a multivariate regression model that included FV_imb_ and relative strength. In this model, the predictive value for changes in jump height decreased, making the overall model statistically non-significant for PAPE-F (β = 0.65, SE = 0.26, R^2^ = 0.43, p = 0.07) and PAPE-V (β = 0.40, SE = 0.31, R^2^ = 0.16, p = 0.40). However, FV_imb_ itself remained a statistically significant predictor when controlling for relative strength (β = 0.62, SE = 0.25, R^2^ = 0.42, p = 0.03), confirming its unique contribution.

## 4 Discussion

This study aimed to determine whether PAPE effects are influenced by addressing individual strength deficits identified through the analysis of the FV profile. We hypothesized that the PAPE-F would offer greater benefits in a sample of strength-deficient male individuals, while an increased FV_imb_ was expected to be associated with a smaller response to both protocols. The first hypothesis was not confirmed as both protocols effectively enhanced JH, with PAPE-F requiring longer recovery to achieve significant JH improvements. However, individuals with more balanced FV profiles experienced greater improvement following PAPE-F (partially supporting our second hypothesis), while no such relationship was observed for PAPE-V. These results suggest that male individuals with a strength deficit, identified through the FV profile, can obtain comparable acute enhancements in JH using force- and velocity-oriented CA, whereas the force-oriented CA is expected to promote greater values for individuals with low FV_imb_. However, it should be noted that the magnitude of the JH change should be interpreted cautiously due to its proximity to the smallest worthwhile change of our device (SWC = 1.3) (Pueo et al., 2020).

Our results are challenging to directly compare with previous studies due to the lack of research considering the individual FV profile as a factor determining the extent of improvement after CAs. Baena-Raya et al. (2020) did not support the significant role of the FV profile with plyometric CA effects on performance enhancement, which is also in line with our results. The parameters of the FV profile differentiate athletes across various sports and skill levels, show strong associations with several performance metrics, and can be modified through short-term training interventions (Baena-Raya et al., 2022; Nishioka and Okada, 2022). However, it remains unclear whether acute enhancements in performance after CAs can be influenced by alterations in the FV profile. Some related insights can be found in studies examining training interventions based on the FV profile, but the results are not entirely compatible with our findings (Baena-Raya et al., 2022). Kasicki et al. (2024) considered factors contributing to PAPE effects, indicating that individual characteristics can influence post-CA responses and, therefore, require further exploration. Our findings point to the potential role of the F–V profile following force-oriented CA.

The first aim of our analysis was to confirm the effectiveness of both protocols in JH enhancement. Our observations indicate that both types of CA significantly affect the PAPE response, aligning with previous research (Wilson et al., 2013; Spieszny et al., 2022; Kalinowski et al., 2022). However, in some cases, plyometric CA may be more effective than force-oriented CA (Krzysztofik et al., 2023). Specifically, we found that the PAPE-F protocol based on maximal isometric efforts required a longer rest period to achieve peak performance than PAPE-V, with improvements noted at 6 and 9 min after CA. This is consistent with other studies showing that peak performance may occur after extended rest intervals ranging from 8 to 12 min (Gouvêa et al., 2013; Kilduff et al., 2008). Data presented by Chen et al. (2023) also showed that a rest interval of 4–9 min is optimal for the beneficial impact on jump performance. Spieszny et al. (2022) observed improved JH in male team sport players following isometric contractions (pushing against an immovable bar). Peak performance occurred at varying times among individuals—some peaked after 4 min, while others peaked after 8 minutes—highlighting the importance of individualized conditions on the PAPE response. Some individuals may require longer rest intervals to maximize the benefits of the CA, highlighting the importance of an individualized approach to rest period selection. Therefore, the question regarding the individual FV profile seems justified. Our results suggest that after PAPE-V, individuals may begin to improve jump performance earlier. Studies have shown that plyometric exercises can induce improvements in subsequent vertical jump performance. Barreto et al. (2023) found that a set of jump exercises enhances subsequent vertical jumps. Similarly, Tobin and Delahunt (2014) reported that professional rugby union players exhibited improved performance in countermovement jumps (CMJs) 1–5 min after performing multiple sets of plyometric CAs. However, their study did not observe effects beyond 5 min, whereas our study noted performance enhancement even after 9 min. The PAPE effect is associated with increased nervous system activation alongside limited muscle energy expenditure during plyometric activities with the involvement of the same muscle groups and similar neuromuscular activation patterns during CA (Seitz and Haff, 2016; Blazevich and Babault, 2019). Both protocols met these conditions; however, PAPE-F may have induced greater fatigue than PAPE-V, necessitating a longer recovery period and thereby delaying improvements in jump performance (French et al., 2003).

Reductions in FV_imb_ have been shown to enhance jump performance (Escobar Álvarez et al., 2020). Jiménez-Reyes et al. (2017) further underscored the value of addressing FV_imb_ to enhance jump abilities, highlighting its potential as a valuable variable in designing CA tailored to optimize explosive performance. Our findings align partially with those observations as individuals with a more balanced FV profile demonstrated greater improvements following the PAPE-F protocol, suggesting that force-oriented interventions may be more effective for those with smaller imbalances. Petridis et al. (2021) also supported the FV profile as a factor related to physical performance. The meta-analysis conducted by Liu et al. (2024) showed that resistance exercises as CAs significantly enhanced both jumping and sprinting performance compared to plyometric or mixed exercises, which are also effective in eliciting performance enhancement. These findings highlight the superior effectiveness of resistance exercises for acute performance improvements in athletes. Thus, our results did not provide clear differences between both types of CA. The effectiveness of PAPE has been linked to the intensity of the stimulus, with higher intensities resulting in more optimal PAPE responses (Esformes and Bampouras, 2013; Kasicki et al., 2024), but individuals with greater strength are more responsive to higher intensities (Seitz and Haff, 2016). This aligns partially with findings from our study showing that male individuals with less strength deficiency were more likely to achieve improvements in JH. Exercises involving maximum isometric contractions (as in PAPE-F) have a great impact on activating motor units associated with force generation (Comfort et al., 2022). Individuals with lower FV_imb_ (a more balanced force–velocity profile) can better utilize their existing strength capacities, leading to more effective adaptation to a stimulus provided with maximal isometric contraction (Fischer and Paternoster, 2024). On the other hand, in PAPE-V, assisted jumps, which rely on the stretch-shortening cycle (SSC), are key for jumping actions (Tomalka et al., 2021; Tillin et al., 2009). Additionally, the complexity of plyometric movements can challenge inexperienced participants. Tobin and Delahunt (2014) found superior PAPE effects with exercises like hurdle and depth jumps in highly trained rugby players, whereas Dello Iacono et al. (2016) reported no significant PAPE in less experienced adolescent athletes, highlighting the role of proficiency in exercise execution. The abovementioned study by Baena-Raya et al. (2020), despite volume of plyometric CA, did not find clear associations between the FV profile and changes in performance after the PAPE protocol. FV_imb_ may not have had a significant impact here because movement velocity is not as closely linked to maximal strength capabilities but rather to coordination and muscle viscoelastic properties (Tillin et al., 2009; Flanagan and Comyns, 2008; Kubo et al., 2021). However, the role of FV profile in plyometric CA effects cannot be totally excluded, and further examination should be performed.

This study has certain limitations that warrant consideration and should be addressed in future studies. One significant limitation was that we did not include participants with velocity deficits, which limits the ability of the findings for participants across the full spectrum of force–velocity imbalances and their impact on PAPE outcomes. We did not employ force plates, which would likely have offered even greater measurement accuracy. In addition, a larger sample size would be required to draw more reliable conclusions in regression analysis. The randomization process was, in part, not satisfactory due to some differences in baseline characteristics (*F*_0_ and *P*_max_). Although our results indicate clear trends in terms of force-oriented CA, uncertainty exists due to the limited number of observations. Although our study follows a typical procedure for PAPE assessment (Xu et al., 2025), increasing the number of jump trials at each time point could yield more precise results, thereby enabling a more accurate evaluation of changes in jump height (Claudino et al., 2017), particularly in the context of the SWC value of the used device (Pueo et al., 2020). Furthermore, the absence of female participants restricts the applicability of the findings to male athletes only, leaving an important gap in understanding gender-specific responses to PAPE protocols. Another limitation was the relatively short measurement time for assessing JH following the conditioning activities. Longer-term monitoring could provide more insights into the time course of PAPE effects and potential delayed benefits of the interventions and associations with the FV profile. The study also focused solely on JH as the performance outcome, excluding other relevant parameters such as sprint performance and power output, which could provide a more comprehensive understanding of the effectiveness of the PAPE protocols. Despite these limitations, this study is the first to explore the interaction between individual force–velocity profiles and PAPE protocols, offering novel insights into how targeted interventions may influence performance outcomes. Future studies should aim to include a more diverse population, assess a broader range of performance metrics, and explore the long-term impacts of tailored PAPE protocols to fully understand their effectiveness and applicability.

## 5 Conclusion

Both the PAPE-F and PAPE-V protocols effectively enhanced JH in following minutes after CA (ES = 0.32–0.33 and ES = 0.24–0.36, respectively) in strength-deficient participants according to the FV profile. The PAPE-F protocol was more beneficial for participants closer to the optimal force–velocity relationship, while no such observation was made after the PAPE-V protocol. These findings suggest that the FV profile may contribute to PAPE effects, emphasizing the importance of tailoring force-oriented CA protocols to address individual force–velocity imbalances. Significant force deficits may result in a diminished response to force-oriented PAPE protocols, highlighting the need for targeted strength development strategies to effectively address force−velocity imbalances.

## Data Availability

The raw data supporting the conclusions of this article will be made available by the authors, without undue reservation.
